# Supplementary figures and images for: Chromatin and Transcriptional Response to Loss of TBX1 in Early Differentiation of Mouse Cells
Source: Front Cell Dev Biol. 2020 Sep 8;8:571501. doi: 10.3389/fcell.2020.571501 (PMC7505952; doi:10.3389/fcell.2020.571501)

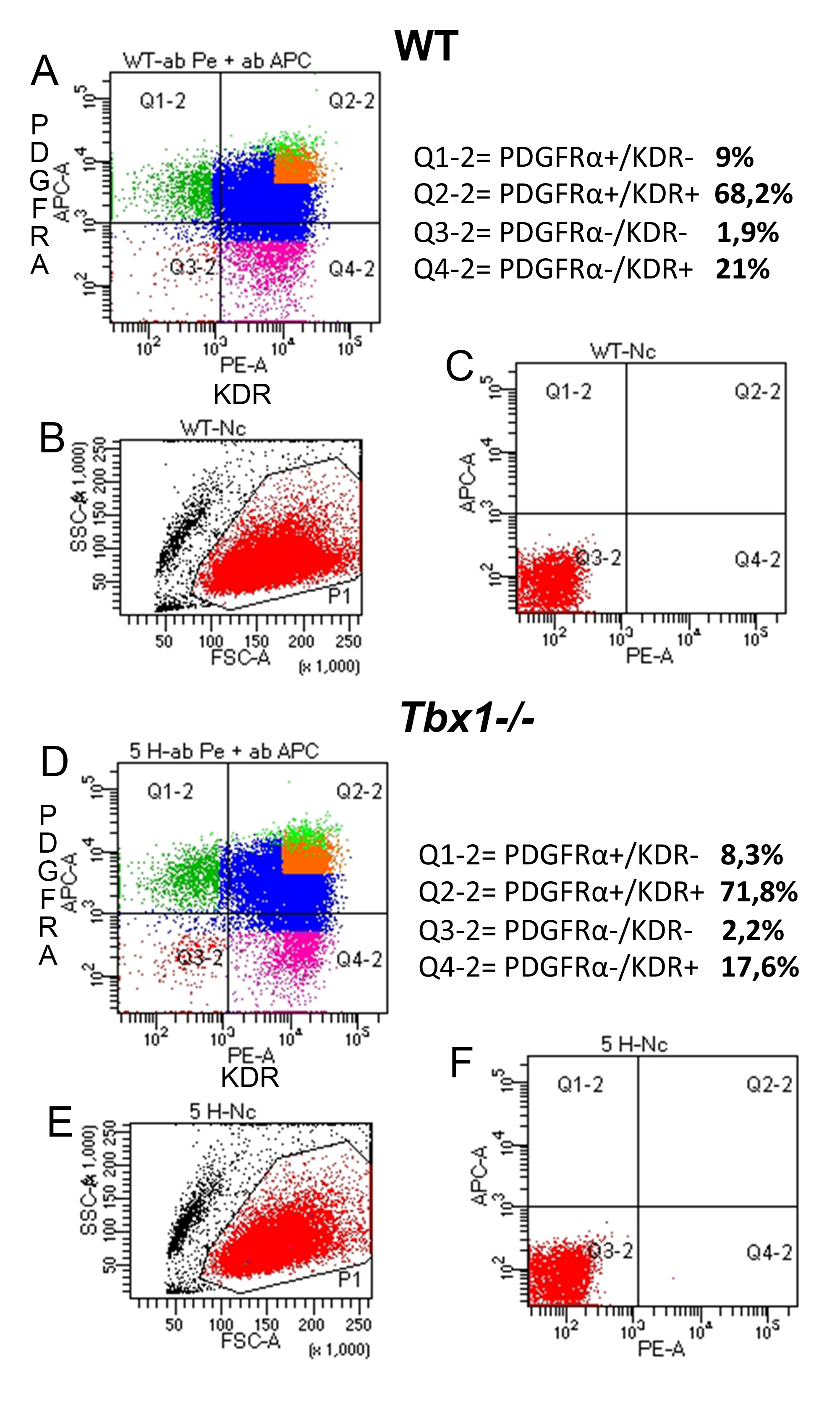

Supplement: FIGURE S1 — Analysis of surface markers PDGFRA and KDR expression in Tbx1+/+ and Tbx1–/– mESC lines at day 4 of differentiation. (A,D) Tbx1+/+ and Tbx1–/– cells labeled by primary antibodies PDGFRA (APC-A) and KDR (PE-A). 4 fractions were identified: Q1-2 = PDGFRA+/KDR−; Q2-2 = PDGFRA+/KDR+; Q4-2 = PDGFRA−/KDR+; Q3-2 = PDGFRA-/KDR−; (B,C,E,F) Negative controls (NC) are cells not incubated with primary antibodies. [file Image_1.JPEG]

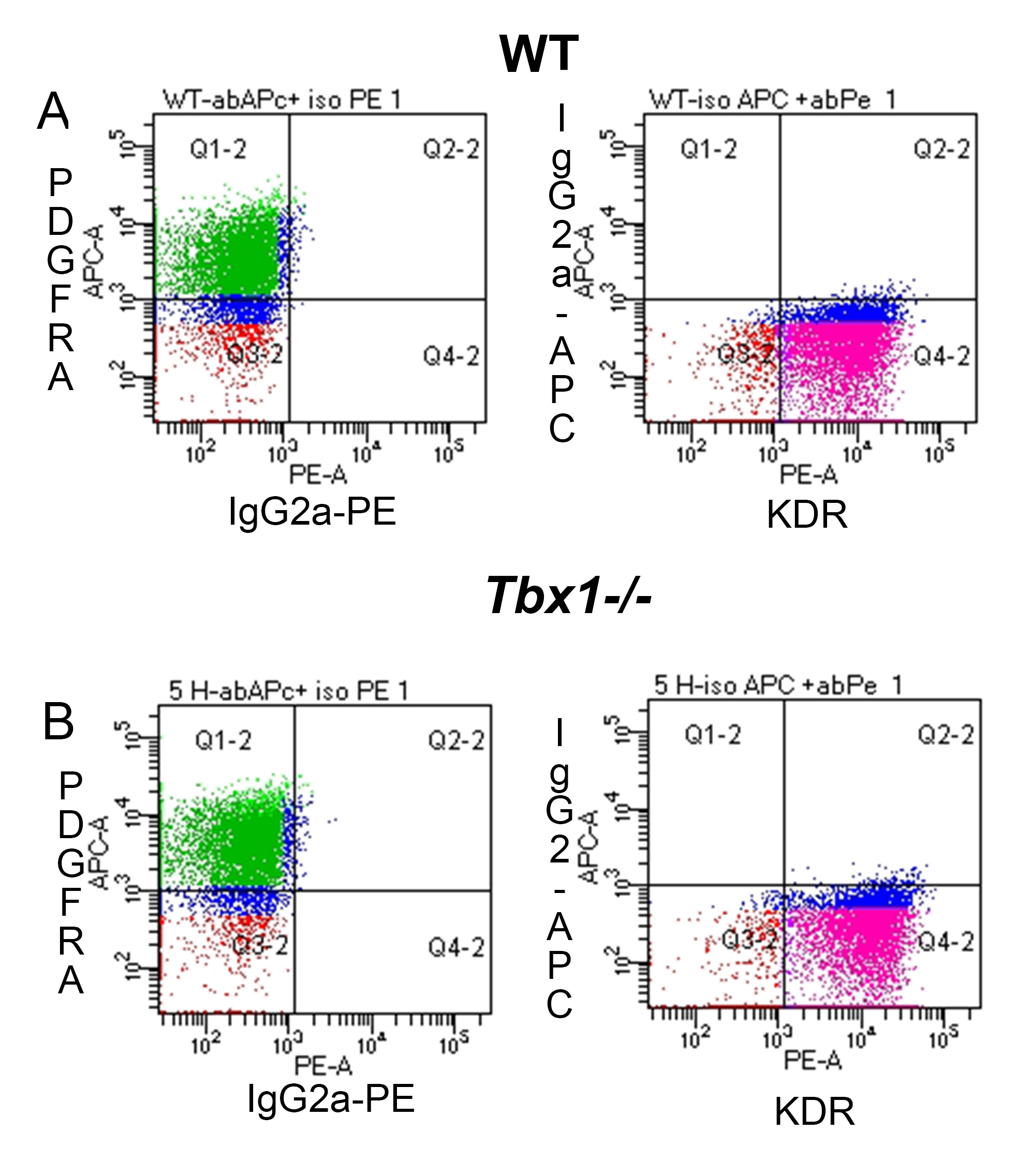

Supplement: FIGURE S2 — Plots showing FACS controls. (A) Tbx1+/+ cells labeled by PDGFRA-APC antibody and isotype control-PE (left); and by isotype control-APC and KDR-PE antibodies (right); (B) Tbx1–/– cells labeled by PDGFRA-APC antibody; and isotype control-PE (left), by isotype control-APC and KDR-PE antibodies (right). [file Image_2.JPEG]

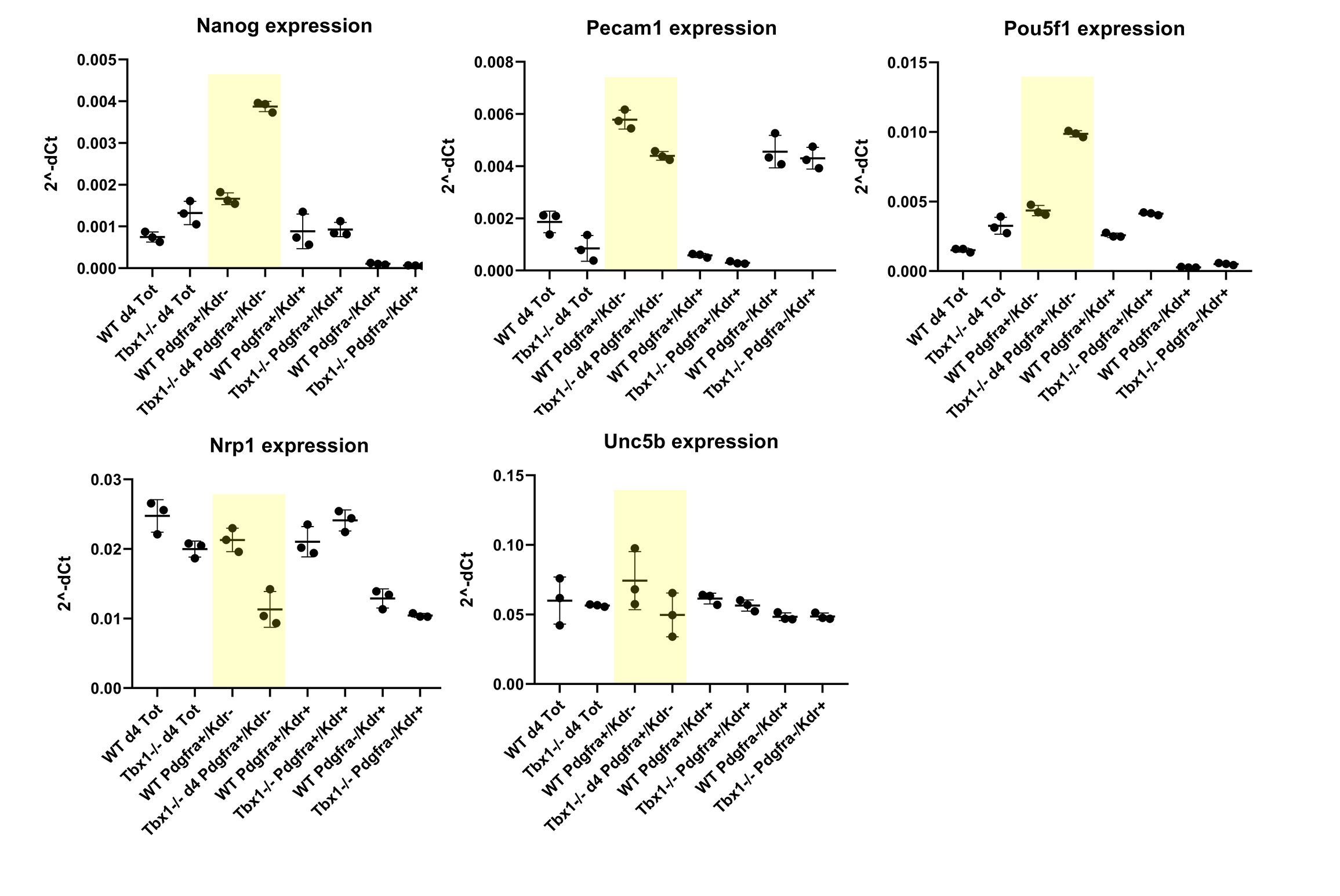

Supplement: FIGURE S3 — Plots showing gene expression assays of five genes in differentiating mESCs. These genes were identified as differentially expressed by RNA-seq in sorted PDGFRA+; KDR− cells. Quantitative real time PCR in unsorted cells at D4 (WT d4 tot. and Tbx1–/– d4 tot.) and in sorted subpopulations showed that the clearest differential expression is only detected in the PDGFRA+; KDR− subpopulation (data shaded in yellow) for all genes tested. [file Image_3.JPEG]
